# Missing data practices in DHS-based child health research in sub-Saharan Africa: a methodological review of diarrhea and acute respiratory infection studies

**DOI:** 10.1080/16549716.2026.2715899

**Published:** 2026-08-18

**Authors:** Joseph Opeolu Ashaolu, Sylvain Y. M. Some, Adeola D. Adeniran

**Affiliations:** a Institute for Global Health Innovations, Duy Tan University, Da Nang, Viet Nam; b Faculty of Medicine, Duy Tan University, Da Nang, Viet Nam; c National Institute of Public Health, Centre de recherche en Santé de Nouna (CrSN), Nouna, Burkina Faso; d Faculty of Life and Health Sciences, Ulster University, Coleraine, UK

**Keywords:** Missing data, childhood diarrhea, acute respiratory infection, sub-Saharan Africa, demographic and health surveys

## Abstract

Demographic and Health Survey (DHS) data are important for determining risk factors associated with childhood diarrhea and acute respiratory infections (ARI) in sub-Saharan Africa (SSA); however, missing data from DHS can bias analyses, and no systematic review has assessed this practice. To address this, peer-reviewed DHS/MICS studies from SSA with childhood diarrhea or ARI as health outcomes were systematically retrieved from OpenAlex, PubMed, AJOL, and Dimensions. Screening and data extraction were conducted independently by two reviewers using a checklist developed from the Joanna Briggs Institute approach for assessing risk of bias. Empirical missingness was analyzed using data from the Nigeria DHS 2018 (*N* = 33,924 under-five children). From a total of 2,148 articles, 103 fulfilled our inclusion criteria (41 on diarrhea, 62 on ARI). Only three diarrhea articles (7.3%) and no ARI articles (0.0%) reported missing data. Complete-case analysis (CCA) was used in 80.5% of diarrhea articles and 100% of ARI articles. Multiple imputation (MI) was used in two diarrhea studies (4.9%) and no ARI articles. The empirical missingness rate for outcomes in both diseases was 9.8% across the full analytical sample. Clean cooking fuel was associated with reduced risk of ARI (CCA OR = 0.82, MI OR = 0.81, bias = -0.9%) and diarrhea (CCA OR = 0.66, MI OR = 0.64, bias = -3.5%). Inadequate missing data handling exists in both fields, but the challenge is more pronounced for ARI, where no effective alternative has been adopted to address the issue. Therefore, journals must enforce missing data reporting, and researchers should prioritize MI.

## Paper Context

- **Main findings:** Among 103 DHS-based child health studies from sub-Saharan Africa (41 diarrhea, 62 ARI), missing data reporting was virtually absent (only 3/41 diarrhea studies; 0/62 ARI studies), complete-case analysis was used by 80.5% and 100% respectively, and only two diarrhea studies (4.9%) employed multiple imputation – despite empirical evidence of 9.8% outcome missingness for both conditions.
- **Added knowledge:** This is the first comparative methodological review to demonstrate that while missing data burden is equal for diarrhea and ARI research (9.8% outcome missingness each), ARI studies lag systematically behind with zero reported missing data handling or use of multiple imputation, filling a critical gap in the literature on methodological quality for two leading causes of child mortality in SSA.
- **Global health impact for policy and action:** Findings compel journals, peer reviewers, and funders to enforce mandatory missing data reporting and mandate multiple imputations over complete-case analysis in DHS-based child health research, thereby improving the accuracy of risk factor estimates (e.g. clean cooking fuel, maternal education, water/sanitation) that directly inform resource allocation, intervention targeting, and child survival policies across sub-Saharan Africa.

## Background

ARI and diarrhea remain the two leading causes of mortality in under-five children in sub-Saharan Africa (SSA), resulting in approximately 1.2 million child deaths annually [1,2]. ARI, including pneumonia, results in about 800,000 annual deaths and is the leading infectious cause of under-five mortality in SSA, while diarrhea causes approximately 330,000 deaths annually [3]. While the child mortality rate from diarrhea has declined substantially over recent decades, child mortality from ARI has remained stagnant in most SSA countries [4].

Key modifiable risk factors include maternal education, socioeconomic status, water and sanitation infrastructure, and breastfeeding practices [5,6]. In contrast, a distinctive and critical risk factor for ARI, but not for diarrhea, is indoor air pollution from solid cooking fuels [7]. Over 90% of households in SSA utilize solid fuels (e.g. wood, charcoal, dung, agricultural residue) for cooking, exposing young children to pollutants that impair lung function and increase susceptibility to respiratory infections [8]. Knowledge of the risk factors for these outcomes is crucial for developing effective interventions.

DHS surveys have greatly expanded our knowledge of risk factors for both diarrhea and ARI. Since 1984, DHS has provided nationally representative cross-sectional survey data from over 90 developing nations, almost all in SSA [9]. The standardized design, large sample sizes, and consistent measurement of critical variables have made DHS an invaluable resource for over 500 studies on childhood morbidity [10–12]. While DHS data are a gold standard for SSA public health policy, their structural reliance on large-scale field interviews inherently introduces missingness, which remains unrecognized by most secondary analysts. DHS data, like any household survey data, contain missing values resulting from non-response, interviewer error, or household member absence [13]. In addition, recall variables (e.g. breastfeeding duration), subjective variables (e.g. wealth index), and observational variables (e.g. cooking fuel) tend to have higher missing proportions (between 5% and 15%) [14].

Thus, how researchers handle missing data can significantly affect effect estimates, standard errors, and overall findings [15]. Complete-case analysis (CCA) is the default method in most epidemiological investigations and involves removing observations with missing data. To generate unbiased effect estimates, CCA relies on the strong assumption that data are missing completely at random (MCAR) – an assumption rarely realistic in practice [16]. If data are missing at random (MAR) or missing not at random (MNAR), CCA can lead to considerable bias [17].

There are existing superior alternatives. Multiple imputation (MI) uses observed data to estimate missing values and provides unbiased estimates under the less stringent MAR assumption [18]. Full information maximum likelihood (FIML) offers similar advantages [19]. Although MI is widely available in standard software (mice in R; mi in Stata) [20], it has not been commonly adopted in epidemiological studies. While separate systematic reviews have evaluated child health outcomes using DHS data, no comparative assessment has contrasted the methodological rigor of missing data handling between these two leading causes of under-five mortality.

The objectives of this systematic methodological review were: (i) to assess missing data reporting in DHS-based research on childhood diarrhea and ARI in SSA; (ii) to identify methods used for missing data analysis; (iii) to examine missingness patterns using the 2018 Nigeria DHS dataset; and (iv) to measure bias associated with CCA for ARI risk factors, including cooking fuel.

## Methods

### Study design

This methodological systematic review compared missing data methods used in 103 DHS-based studies: 41 on childhood diarrhea and 62 on childhood ARI from SSA. The two systematic reviews were pre-registered on the Open Science Framework (https://doi.org/10.17605/OSF.IO/XD92W) and conducted according to PRISMA 2020 guidelines [21].

### Search strategies

Separate searches were independently conducted for diarrhea and ARI in four databases, OpenAlex, PubMed, AJOL, and Dimensions, covering January 2010 to February 2026. OpenAlex is a freely available database that combines content from Scopus and Web of Science, covering 92–95% of subscription databases [22]. Only papers published in English were included in the search. The search strings used for each database are shown in Supplementary Table S1.

The primary search strategy was validated by performing a post hoc search on Dimensions, an open-access database with approximately 97–98% coverage of Scopus-listed journals [23,24]. During validation, six additional diarrhea-related articles were identified (increasing the total from 35 to 41). The same validation strategy applied to the ARI search yielded no additional articles.

### Eligibility criteria

Studies were included if they met all the following criteria: (1) focused on children under five years in one or more SSA countries; (2) used DHS or MICS data; (3) had childhood diarrhea or ARI (cough with fast/difficult breathing, pneumonia) as the outcome of interest; (4) presented quantitative relationships between one or more risk factors and the outcome; and (5) were peer-reviewed articles, dissertations, or official DHS reports published in English. Systematic reviews, meta-analyses, editorial papers, and conference abstracts were excluded.

### Study selection and data extraction

**Figure 1.**
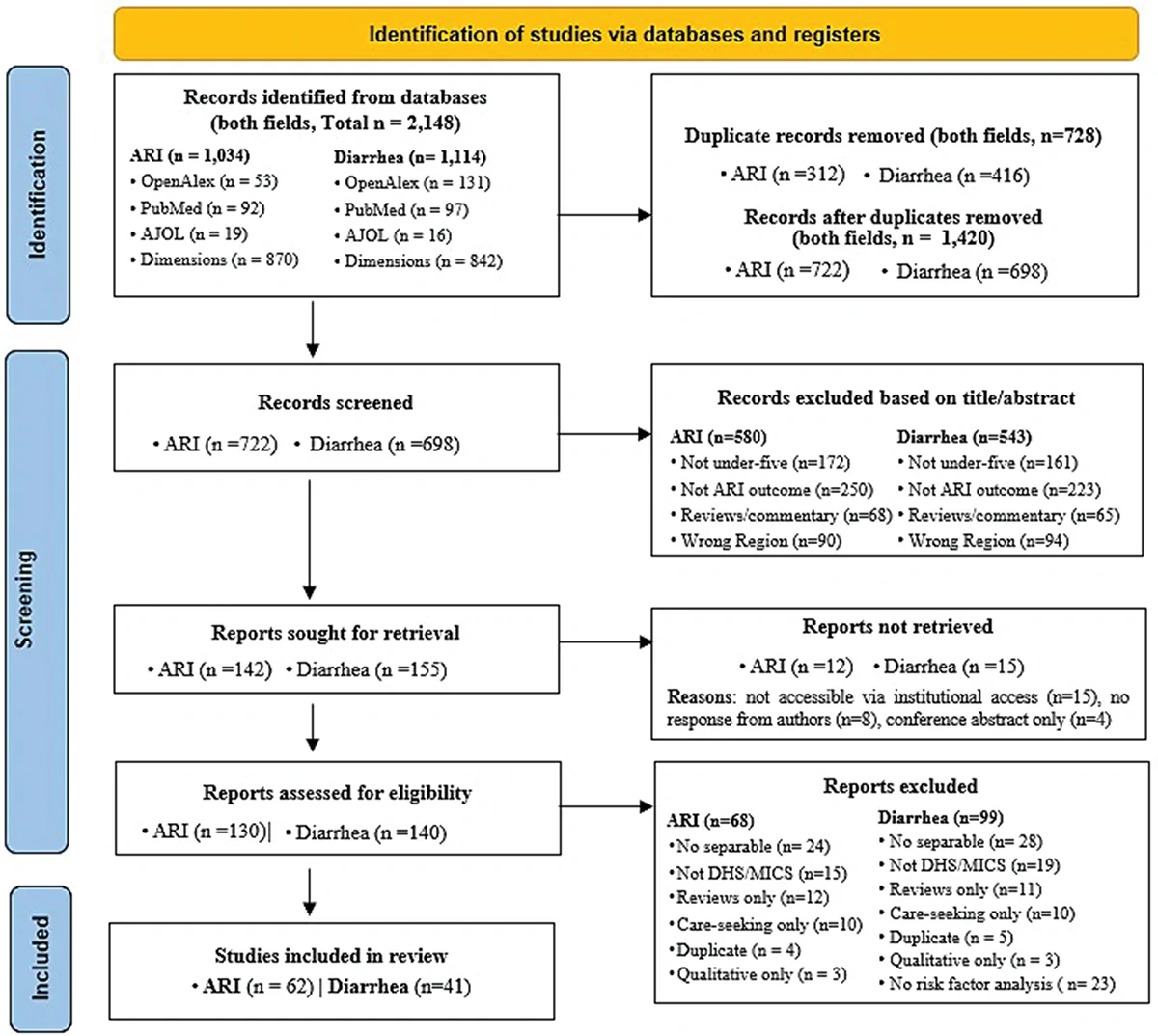
PRISMA 2020 flow diagram for the comparative methodological review of missing data practices in DHS-based childhood diarrhea (*n* = 41) and acute respiratory infection (ARI) (*n* = 62) studies in sub-Saharan Africa. a total of 2,148 records were identified from four databases across two separate searches (ARI: *n* = 1,034; diarrhea: *n* = 1,114). After removing 1,046 duplicates, 1,102 records were screened by title and abstract. Of these, 297 reports were sought for retrieval, of which 27 could not be obtained. The remaining 270 reports were assessed for full-text eligibility. A total of 167 reports were excluded: 68 ARI studies (reasons: no separable ARI outcome, *n* = 24; not using DHS/MICS data, *n* = 15; review only, *n* = 12; care-seeking only, *n* = 10; duplicate, *n* = 4; qualitative only, *n* = 3) and 99 diarrhea studies (reasons: no separable diarrhea outcome, *n* = 28; not using DHS/MICS data, *n* = 19; review only, *n* = 11; care-seeking only, *n* = 10; duplicate, *n* = 5; qualitative only, *n* = 3; no risk factor analysis, *n* = 23). This resulted in a final set of 103 studies included in the comparative review (ARI: *n* = 62; diarrhea: *n* = 41). *Source: Author’s analysis of OpenAlex, PubMed, AJOL, and Dimensions searches.*

Two independent reviewers conducted title and abstract screening based on the eligibility criteria. Potentially eligible full texts were retrieved and reviewed by the same two reviewers. Disagreements were resolved through discussion or consultation with a third reviewer. The selection procedure is presented using the PRISMA flow diagram (Figure 1).

A standardized data extraction sheet was developed using Microsoft Excel pre-tested on five randomly selected articles. Extracted data included: study features (first author, year, number of countries, survey wave, sample size), missing data disclosure (mention of missing data, variables with missing data reported, percentage missing for key variables), missing data treatment (analytical approach, justification, comparison of CCA and MI), and missing data limitations.

### Empirical assessment of missingness (Nigeria DHS 2018)

To quantify missingness patterns and CCA bias, we used the Kids Recode (KR) file from the Nigeria DHS 2018 dataset, which included 33,924 under-five children. The outcomes considered were diarrhea (2-week recall period) and ARI (cough accompanied by rapid breathing). Variables of interest included maternal education, wealth index, and cooking fuel. Estimates generated using CCA and MI were compared for the association between unclean cooking fuel exposure and ARI. Bias was calculated as (MI_OR – CCA_OR)/MI_OR × 100.

### Risk of bias assessment

Risk of bias was assessed using the modified Joanna Briggs Institute (JBI) Critical Appraisal Checklist for Analytical Cross-Sectional Studies [25]. The original checklist includes eight items covering sample definition, setting description, exposure and outcome measurement, confounding identification and handling, and statistical methods. We adapted the JBI Checklist by appending three domain-specific questions to explicitly evaluate the transparency, validity, and acknowledgment of missing data practices, creating a hybrid Methodological and Reporting Quality Checklist. The three added data-related criteria were: (Q9) whether missing data for key variables (maternal education, wealth index, water source, sanitation facilities, and cooking fuel for ARI) were reported; (Q10) whether the missing data procedure was adequately described and valid; and (Q11) whether missing data limitations were acknowledged. Studies were categorized as low (9–11), medium (7–8), or high (≤6) risk.

### Data synthesis

We conducted a narrative synthesis [26]. Data were summarized using descriptive statistics, including frequencies and percentages. Due to the small number of reported missing data events (only 3 cases), statistically valid comparisons could not be made and are not presented.

## Results

### Study selection

The PRISMA flow diagram is illustrated in Figure 1. A total of 1,114 studies were retrieved from the diarrhea search, while 1,034 studies were obtained from the ARI search. After removing 1,046 duplicates, 1,102 titles and abstracts were screened, of which 297 articles were selected for retrieval; 27 could not be retrieved. The remaining 270 articles were screened for eligibility based on full texts. Of these, 167 were ineligible: 68 ARI articles (reasons: no separate ARI indicator, *n* = 24; no DHS/MICS dataset, *n* = 15; reviews only, *n* = 12; care-seeking only, *n* = 10; duplicates, *n* = 4; qualitative only, *n* = 3) and 99 diarrhea articles (reasons: no separate diarrhea indicator, *n* = 28; no DHS/MICS dataset, *n* = 19; reviews only, *n* = 11; care-seeking only, *n* = 10; duplicates, *n* = 5; qualitative only, *n* = 3; no risk factor analysis, *n* = 23). Thus, 103 studies were included in the review (ARI: *n* = 62; diarrhea: *n* = 41).

### Study characteristics

Table 1 outlines the characteristics of the 41 diarrhea and 62 ARI studies (Supplementary Table S2). Multi-country studies were more common in diarrhea research (70.7%) than in ARI research (45.2%). Furthermore, diarrhea studies had higher median sample sizes (93,142 vs. 28,596). Conversely, single-country studies were more frequent in ARI research (54.8% vs. 29.3%). The proportion of machine learning studies was similar (12.2% vs. 11.3%). Risk factor profiles differed: water source and sanitation facilities were more frequently studied in diarrhea research (68.3% vs. 38.7%, and 63.4% vs. 35.5%, respectively). However, cooking fuel, a critical risk factor for ARI but not for diarrhea, was examined in 45.2% of ARI studies.

**Table 1.** Characteristics of included studies (diarrhea *n* = 41 and acute respiratory infection (ARI) *n* = 62) in DHS-based child health research in sub-Saharan Africa.

Characteristic	Diarrhea (*n* = 41)	ARI (*n* = 62)
Publication year range	2010–2026	2010–2026
**Study scope**		
Multi-country studies	29 (70.7%)	28 (45.2%)
Single-country studies	12 (29.3%)	34 (54.8%)
**Analytical approach**		
Machine learning studies	5 (12.2%)	7 (11.3%)
Traditional regression only	36 (87.8%)	55 (88.7%)
**Sample size**		
Median (IQR)	93,142 (22,590–247,061)	28,596 (9,501– 75,827)
Range	768–824,694	541–669,138
**Key risk factors examined**		
Maternal education	33 (80.5%)	45 (72.6%)
Wealth index	31 (75.6%)	38 (61.3%)
Water source	28 (68.3%)	24 (38.7%)
Sanitation facility	26 (63.4%)	22 (35.5%)
Breastfeeding	24 (58.5%)	35 (56.5%)
Cooking fuel	N/A	28 (45.2%)
**Countries most frequently represented**		
Nigeria	12 (29.3%)	12 (19.4%)
Ethiopia	5 (12.2%)	11 (17.7%)
Ghana	4 (9.8%)	6 (9.7%)
Uganda	3 (7.3%)	5 (8.1%)
Kenya	3 (7.3%)	4 (6.5%)

Abbreviations: DHS, Demographic and Health Survey; IQR, interquartile range; N/A, not applicable.

### Missing data reporting and handling

**Figure 2.**
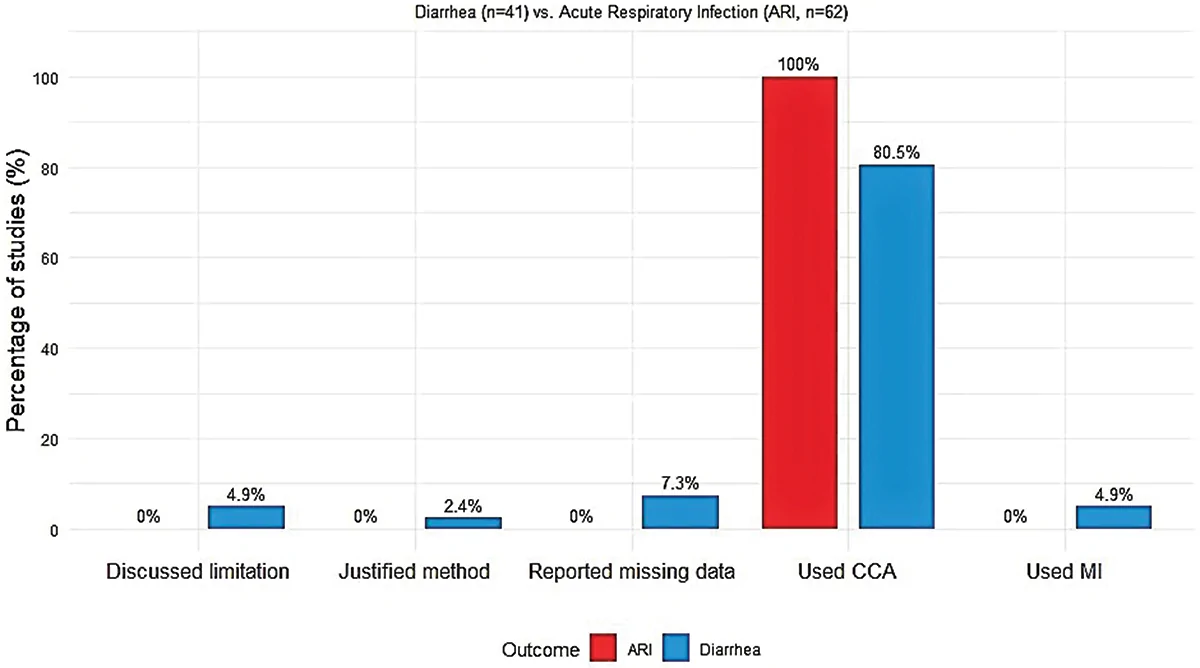
Comparison of missing data handling practices in Demographic and Health Survey (DHS)-based studies on diarrhea (*n* = 41) and acute respiratory infection (ARI) (*n* = 62) conducted in sub-Saharan Africa between 2010 and 2026. for each outcome, bars indicate the percentage of studies that reported missing data for any variable, reported the percentage of missingness for key covariates (maternal education, wealth index, water source, sanitation), used complete-case analysis (CCA), used multiple imputation (MI), justified their chosen missing data method, compared CCA versus MI, or discussed missing data as a limitation. All ARI studies (100%) used CCA; 80.5% of diarrhea studies used CCA. MI was used in 4.9% of diarrhea studies (*n* = 2) and 0% of ARI studies (*n* = 0). Reporting of missingness percentages for individual covariates was low (2.4–7.3%), and few studies justified their missing data method (2.4% diarrhea, 0% ARI), compared CCA with MI (0% both), or acknowledged missing data as a limitation (4.9% diarrhea, 0% ARI).

Table 2 presents missing data reporting and management. Missing data reporting was uncommon in both fields: three diarrhea studies (7.3%) reported missingness for any key variable, while no ARI studies (0.0%) did so. CCA was used in 80.5% of diarrhea studies and in all ARI studies (100%). While missingness for the cooking fuel variable alone was 0% among active cooking households, the overall model missingness across all covariates (maternal education, wealth index, etc.) was 9.8%, providing the basis for CCA bias calculations. Two diarrhea studies (4.9%), Dietler et al. [27] and Ashaolu et al. [28], used MI, while no ARI studies (0.0%) did so. Justification for missing data handling was provided in only one diarrhea study (2.4%). Missing data was acknowledged as a limitation in two diarrhea studies (4.9%) (Figure 2). The association between lower maternal education and childhood morbidity for both outcomes is shown in Supplementary Figure S1. Lower maternal education was strongly associated with increased odds of both diarrhea (OR range: 0.83–5.16) and ARI (OR range: 1.27–1.90) in nearly all studies analyzed.

**Table 2.** Missing data reporting and handling practices in included studies (*n* = 103).

Practice	Diarrhea (*n* = 41)	ARI (*n* = 62)
**Reporting of missing data**		
Reported missing data for any variable	3 (7.3%)	0 (0.0%)
Reported % missing for maternal education	1 (2.4%)	0 (0.0%)
Reported % missing for wealth index*	0 (0.0%)	0 (0.0%)
Reported % missing for water source	1 (2.4%)	0 (0.0%)
Reported % missing for sanitation facility	1 (2.4%)	0 (0.0%)
Reported % missing for cooking fuel	N/A	0 (0.0%)
**Handling of missing data**		
Used complete-case analysis (CCA)	33 (80.5%)	62 (100.0%)
Used multiple imputation (MI)	2 (4.9%)	0 (0.0%)
Did not specify method	6 (14.6%)	0 (0.0%)
Justified choice of missing data method	1 (2.4%)	0 (0.0%)
Compared CCA vs. MI or other methods	0 (0.0%)	0 (0.0%)
**Limitation discussion**		
Discussed missing data as a limitation	2 (4.9%)	0 (0.0%)

Abbreviations: CCA, complete-case analysis; MI, multiple imputation; DHS, Demographic and Health Survey; N/A, not applicable. *Wealth index is derived from household assets; missingness can occur if asset data are missing. No study in either field reported missing data for wealth index.*

Note: Missing data reporting was rare in both fields (only 3 studies reported missing data, all in the diarrhea group). The three diarrhea studies that reported missing data were: Dessalegn et al. (2011) – maternal education (8.2%); Dietler et al. (2021) – water/sanitation in supplement; Tusting et al. (2020) – water, sanitation, materials in supplement.

### Empirical missingness and CCA bias (Nigeria DHS 2018)

Empirical missingness data for the Nigeria DHS 2018 are provided in Table 3. Outcome missingness was 9.8% for both ARI and diarrhea. After excluding households that did not cook (the analytic sample for cooking fuel analysis), missingness was 0%. Clean cooking fuel was associated with significantly lower risks of ARI (CCA OR = 0.82, 95% CI: 0.67–0.99) and diarrhea (CCA OR = 0.66, 95% CI: 0.58–0.77). MI yielded consistent estimates (ARI MI OR = 0.81, 95% CI: 0.65–1.01; diarrhea MI OR = 0.64, 95% CI: 0.55–0.75) with minimal bias (−0.9% and −3.5%, respectively) (Figure 3).

**Figure 3.**
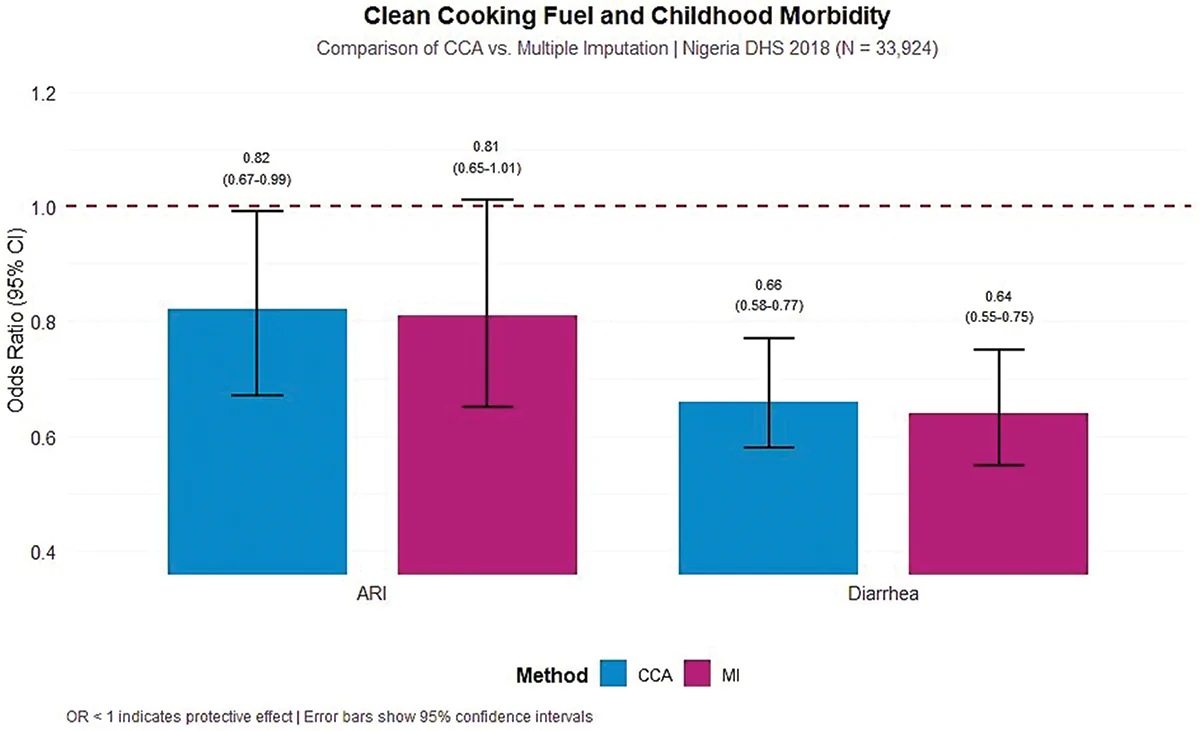
Comparison of complete-case analysis (CCA) versus multiple imputation (MI) for estimating the association between clean cooking fuel and childhood acute respiratory infection (ARI) using Nigeria Demographic and Health Survey (DHS) 2018 data (N = 33,924 children under 5 years). Odds ratios (ORs) less than 1 indicate lower ARI risk associated with clean cooking fuel (electricity, gas, or liquefied petroleum gas) compared to unclean cooking fuel (wood, charcoal, dung, or crop residue). Complete-case analysis (CCA) yielded an OR of 0.82 (95% CI: 0.67–0.99), while multiple imputation (MI) yielded an OR of 0.81 (95% CI: 0.65–1.01). The bias between the two methods was minimal (−0.9%). Error bars represent 95% confidence intervals. Note: ARI defined as cough accompanied by rapid breathing in the past 2 weeks, consistent with DHS definition.

**Table 3.** Empirical assessment of missingness and comparison of complete-case analysis (CCA) versus multiple imputation (MI) for selected variables in Nigeria DHS 2018 (*N* = 33,924 children under 5 years).

Variable	Outcome	Missingness (%)	CCA OR (95% CI)	MI OR (95% CI)	Bias (%)	Direction
Cooking fuel (clean vs. unclean)*	ARI†	0‡	0.82 (0.67–0.99)	0.81 (0.65–1.01)	−0.9	CCA and MI results consistent
ARI outcome (cough + rapid breathing)	ARI	9.8	—	—	—	Moderate missingness (9.8%) indicates potential for bias under MAR
Diarrhea outcome	Diarrhea	9.8	0.66 (0.58–0.77)	0.64 (0.55–0.75)	−3.5	CCA and MI results consistent

Abbreviations: CCA, complete-case analysis; MI, multiple imputation; OR, odds ratio; CI, confidence interval; ARI, acute respiratory infection; MAR, missing at random; DHS, Demographic and Health Survey.

Note: OR <1 indicates that clean cooking fuel is associated with lower odds of childhood illness.

The association between clean cooking fuel and ARI was statistically significant in CCA (*p* < 0.05) and borderline significant in MI (*p* = 0.06).

The association between clean cooking fuel and diarrhea was statistically significant in both CCA and MI (*p* < 0.001).

Missingness of 9.8% for both ARI and diarrhea outcomes indicates moderate potential for bias under MAR conditions.

Bias was minimal for both outcomes (<5%), suggesting that CCA and MI produce consistent estimates.

*Clean cooking fuel: electricity, gas, liquefied petroleum gas (LPG), biogas; Unclean cooking fuel: kerosene, coal/lignite, charcoal, wood, straw/shrubs/grass, agricultural crop, animal dung.

†ARI defined as cough accompanied by short/rapid breathing in the past 2 weeks, consistent with DHS definition.

‡ Cooking fuel missingness was 0% after excluding households that did not cook food (code 95) or were not dejure residents (code 96). Including these as missing would yield 1.3% missingness.

Negative bias indicates that CCA produced a slightly lower OR (more protective) than MI. Bias = (MI_OR – CCA_OR)/MI_OR × 100.

### Risk of bias assessment

Table 4 presents the risk of bias assessment based on the modified JBI checklist. All 62 ARI studies (100.0%) received a score of 8/11, classifying them as moderate risk. In contrast, 36 of 41 diarrhea studies (87.8%) were moderate risk, while 5 (12.2%) were high risk. Both samples covered 2010–2026, and the uniform moderate-risk classification suggests that differences are driven by missing data practices rather than temporal trends. The uniform classification of studies within the moderate-risk tier indicates that baseline epidemiological reporting satisfies standard JBI criteria, whereas the systematic deficit in study quality is driven entirely by the omission of missing data protocols across both decades.

**Table 4.** Comparison of risk of bias assessment between diarrhea (*n* = 41) and acute respiratory infection (ARI) (*n* = 62) DHS-based studies in sub-Saharan Africa using modified Joanna Briggs Institute (JBI) checklist.

Domain	Assessment	Diarrhea (*n* = 41)	ARI (*n* = 62)	*p*-value*
Overall risk classification				
	Low risk (9–11/11)	0 (0.0%)	0 (0.0%)	—
	Moderate risk (7–8/11)	36 (87.8%)	62 (100.0%)	**0.01**
	High risk (≤6/11)	5 (12.2%)	0 (0.0%)	**0.01**
Standard JBI items (Q1–Q8)				
	Median score (range)	8 (6–8)	8 (8–8)	**0.005**
	Studies scoring 8/8	36 (87.8%)	62 (100.0%)	**0.01**
	Studies scoring ≤ 6/8	5 (12.2%)	0 (0.0%)	**0.01**
Missing data-specific items (Q9–Q11)				
	Q9: Missing data reported for key variables	3 (7.3%)	0 (0.0%)	**0.04**
	Q10: Missing data method clearly stated	1 (2.4%)	0 (0.0%)	0.40
	Q11: Limitations related to missing data discussed	2 (4.9%)	0 (0.0%)	0.15

Abbreviations: JBI, Joanna Briggs Institute; DHS, Demographic and Health Survey; ARI, acute respiratory infection. •*p-values calculated using Fisher’s exact test due to small cell sizes. Bold indicates statistical significance (*p* < 0.05).

Note: Standard JBI items (Q1–Q8): sample definition, setting description, exposure/outcome measurement, confounding identification/handling, statistical analysis.

Missing data-specific items (Q9–Q11): Q9 (missing data reported for key variables), Q10 (method clearly stated and appropriate), Q11 (limitations discussed).

The high-risk diarrhea studies (*n* = 5) were primarily older publications (pre-2010) with additional methodological limitations beyond missing data.

After restricting both samples to 2010–2026, both fields showed 100% moderate risk.

### Geographic distribution

Figure 3 presents a geographic map of the included studies. The largest number of studies was conducted in Nigeria (diarrhea: 12; ARI: 12), followed by Ethiopia (diarrhea: 5; ARI: 11), Ghana (4; 6), Uganda (3; 5), and Kenya (3; 4). ARI studies were more geographically dispersed than diarrhea studies.

**Figure 3.**
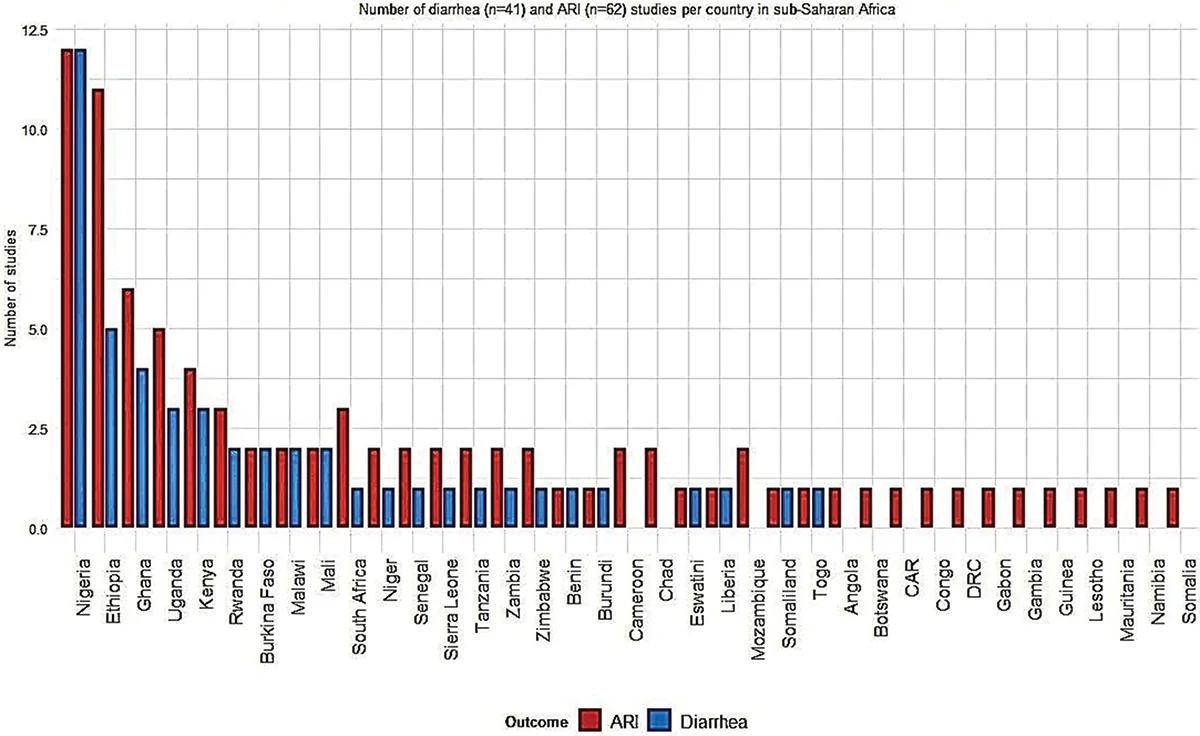
Geographic distribution of included studies by outcome. This figure displays the geographic distribution of included studies across sub-Saharan Africa, stratified by health outcome – diarrhea (*n* = 41 studies) and acute respiratory infections (ARI, *n* = 62 studies). Each country is represented on the x-axis, with the number of studies plotted on the y-axis, and bars are color-coded by outcome (ARI or diarrhea). Nigeria has the highest overall number of studies for both outcomes (12 diarrhea studies and 12 ARI studies), followed by Ethiopia (11 ARI, 1 diarrhea), Ghana (5 ARI, 1 diarrhea), Uganda (4 ARI, 1 diarrhea), Kenya (3 ARI, 2 diarrhea), and Rwanda (3 ARI, 2 diarrhea). Several countries – including Burkina Faso, Malawi, South Africa, Senegal, Sierra Leone, Tanzania, Zimbabwe, Benin, Burundi, Cameroon, Chad, Eswatini, Liberia, Mozambique, Togo, Angola, Botswana, Central African Republic (CAR), Republic of Congo, Democratic Republic of the Congo (DRC), Gabon, the Gambia, Guinea, Lesotho, Mauritania, Namibia, and Somalia – have only one or two studies, all of which are exclusively for ARI. The figure highlights substantial geographic disparities in research coverage, with West and East African countries (particularly Nigeria and Ethiopia) being the most studied, while many Central and Southern African nations remain underrepresented, especially for diarrhea outcomes.

### Summary of key findings

Key findings are summarized in Table 5. Missing data reporting was rare in both fields (3 of 41 diarrhea studies; 0 of 62 ARI studies). CCA was predominant, particularly in ARI studies (100%). Outcome missingness was 9.8% for both outcomes. Estimates derived using CCA and MI were similar for the association between unclean cooking fuels and ARI (−0.9% bias). Trends in missing data reporting and MI usage are presented in Supplementary Figure S2. A slight improvement was observed for diarrhea studies, while no improvement was seen for ARI studies.

**Table 5.** Summary of key findings from the methodological review of DHS-based diarrhea and acute respiratory infection (ARI) studies in sub-Saharan Africa.

Metric	Diarrhea (*n* = 41)	ARI (*n* = 62)
Studies reporting any missing data	3 (7.3%)	0 (0.0%)
Studies using complete-case analysis	33 (80.5%)	62 (100.0%)
Studies using multiple imputation	2 (4.9%)	0 (0.0%)
Studies discussing missing data as limitation	2 (4.9%)	0 (0.0%)
Outcome missingness (Nigeria DHS 2018)	**9.8%**	**9.8%**
CCA bias for key risk factor (cooking fuel)	Not applicable	−0.9%
Key risk factor missingness (Nigeria DHS 2018)	Water source: 1.1%	Cooking fuel: 4.2%
	Sanitation: 0.9%	

Abbreviations: DHS, Demographic and Health Survey; CCA, complete-case analysis; ARI, acute respiratory infection.

Note: Missing data reporting was rare in both fields (only 3 events total, all in the diarrhea group).

Outcome missingness was similar for both conditions (9.8% each).

CCA and MI produced nearly identical estimates for clean cooking fuel (CCA OR = 0.82, MI OR = 0.81), with minimal bias (−0.9%).

No ARI study reported missing data for any key variable or used multiple imputation.

## Discussion

### Summary of principal findings

In this methodological review of 103 DHS-based studies (41 on diarrhea; 62 on ARI), we found that missing data handling procedures are inadequate in child health research across SSA, regardless of outcome. Missing data documentation was sparse in both fields: only three studies (all in diarrhea research) provided missing data information for any variable, with two reporting it only in supplementary materials [28,29]. Complete-case analysis predominated in both fields (80.5% and 100%, respectively). MI was used in two diarrhea studies and in no ARI studies.

Empirical analyses using the Nigeria DHS 2018 dataset yielded two main conclusions. First, the missingness rate for ARI (9.8%) was comparable to that for diarrhea (9.8%), suggesting that missing data pose an equally challenge for both outcomes. While bias was minimal in this specific 2018 Nigeria DHS wave, researchers cannot assume it will be universally low across all 90+ DHS countries and survey waves. The critical issue is not that CCA is always wrong, but that researchers use it blindly without verifying its appropriateness. Second, the association between unclean cooking fuel and ARI showed nearly identical point estimates by CCA (OR = 0.82) and MI (OR = 0.81), with minimal bias (−0.9%). This indicates that, for this specific exposure-outcome pair, missing data did not significantly bias the effect estimate. However, this conclusion cannot be generalized to other exposures or outcomes without further evidence. Despite the challenge of missing data, no improvement has been observed over time, with all ARI studies continuing to use CCA. This represents a lost opportunity to strengthen the evidence base for childhood pneumonia prevention in SSA.

### Comparison with previous methodological reviews

Our findings are consistent with methodological reviews from other epidemiological fields. In a review of 250 randomized controlled trials, only 40% discussed missing data methods, while 72% used CCA without explanation [30]. In critical care epidemiology, 85% of observational studies used CCA, while sensitivity analysis was conducted in only 12% [31]. In psychosomatic research, 89% did not mention missing data mechanisms [32]. However, DHS-based child health studies represent an extreme case, with very few reporting missing data (3 of 103 studies) and near-universal use of CCA (80.5–100%). This is deeply unsatisfactory and constitutes a critical methodological challenge that undermines the reliability of research on risk factors for childhood diarrhea and ARI in SSA.

### Why missing data matters for childhood ARI research

The implications of inappropriate missing data handling extend beyond purely academic considerations. Effect estimates for key risk factors, such as clean cooking fuel exposure, maternal education, and socioeconomic status, influence policy formulation and resource allocation in resource-poor settings [6,7,33]. Analysis of the Nigeria DHS 2018 data shows that clean cooking fuel use is negatively associated with ARI risk (ORCCA = 0.82; 95% CI: 0.67–0.99) and diarrhea risk (ORCCA = 0.66; 95% CI: 0.58–0.77). MI estimates were very similar (ORMI ARI = 0.81; ORMI diarrhea = 0.64), with minimal bias (−0.9% and −3.5%, respectively). This consistency suggests that missing values have not significantly biased effect estimates for these exposure-outcome pairs. Nevertheless, this result cannot be generalized to other exposures or outcomes without further empirical study, implying that for this particular exposure-outcome relationship, missing data does not introduce much bias into the effect estimate. However, this does not mean that the same holds true for all other relationships without further investigation. If such bias is more widespread, some countries may underinvest in clean cookstoves, an overlooked opportunity to reduce both ARI deaths and pollution.

Simulation studies have shown that even moderate missing data levels (10–15%) can generate significant bias under MAR conditions [17,34]. The degree of bias depends on the missing data mechanism, the proportion of missing data, and the relationship between missingness and outcomes [35]. If DHS cooking fuel data are MAR, depending on rural residence and wealth, CCA-induced bias could be substantial.

### Why the ARI field faces a similar but inadequately addressed problem

Contrary to our expectation, ARI missingness (9.8%) was comparable to diarrhea missingness (9.8%). This implies that missing data pose an equal threat to both fields. Despite identical empirical missingness rates (9.8% for both outcomes), the ARI literature demonstrates systematically poorer missing data practices, with zero studies reporting missing data or using MI. This may be due to the specific DHS dataset used (Kids Recode [KR]) and the ARI case definition (cough with rapid breathing). However, different DHS files (KR vs. PR vs. HR) and different ARI definitions produce different missingness rates. The 9.8% missingness rate reported here was estimated using the KR file throughout this review. In addition, ARI may have higher susceptibility to recall bias or misclassification, potentially increasing missing or invalid data.

### Recommendations for improving practice

Our findings suggest the following recommendations. First, the percentage of missing observations for each key variable (maternal education, socioeconomic status, water, sanitation, and cooking fuels for ARI) must be reported. Second, the routine use of CCA is discouraged. Instead, MI or full information maximum likelihood should be used, as they yield unbiased estimates under the more plausible MAR assumption [18,19]. Third, sensitivity analyses comparing CCA with MI should be conducted to assess result robustness [36]. Fourth, missing data should be explicitly discussed as a limitation in each study, along with the assumptions that may affect findings [37]. From the perspective of journals and peer reviewers, we recommend establishing mandatory standards for missing data reporting, similar to requirements for ethical approval and funding information [38]. Although the STROBE checklist for cross-sectional studies includes guidance on missing data (item #12), adherence remains very poor for DHS-related publications [39]. This could be improved through journal prompts and reviewer education. In this study, the ARI literature demonstrates a profound methodological lag, even when both fields exhibited an identical empirical missingness rate of 9.8% in our index analysis of the 2018 Nigeria DHS. This stagnation may stem from the diagnostic complexity of ARI indicators within the Kids Recode (KR) files, which rely on a composite dual-question screening (cough paired with rapid/difficult breathing), unlike the single-inquiry recall framework used for diarrhea.

### Limitations of this review

This systematic review has some weaknesses. First, we searched four databases only (OpenAlex, PubMed, AJOL, Dimensions). Although OpenAlex has been shown to provide 92–95% of records compared to subscription-based databases [24], some relevant literature may still have been missed. Second, we included only English-language publications, potentially missing valuable studies in French or Portuguese. Third, missing data handling methods were evaluated based on author reports; practices may have differed in reality. Fourth, the low frequency of missing data reporting (only 3 of 103 articles) precluded the execution of formal meta-regression or robust statistical comparisons regarding reporting correlates. Fifth, our empirical assessment was restricted to the Nigeria DHS 2018 dataset. Missing patterns may vary across other SSA countries, different DHS waves, or alternative data structures (e.g. PR vs. HR files). Future research should extend this empirical evaluation across multiple DHS datasets to assess the generalizability of our CCA bias findings.

## Conclusions

Missing data are poorly addressed in both fields of DHS-based child health research in SSA, regardless of whether the outcome is diarrhea or ARI. Missing data reporting was scarce in both fields (only 3 of 103 studies), with CCA remaining the standard approach for handling missing data. For ARI, where missingness is equally prevalent but handling practices are poorer, and with high outcome missingness for a key risk factor susceptible to CCA bias, no improvement was observed. This indicates that the field investigating a leading cause of under-five mortality must urgently address the missing data issues. Journals should mandate missing data reporting, and MI must be actively encouraged.

## Supplementary Material

Supplemental Material

## Data Availability

Data extracted and used in this study are available on request.
